# Targeting the Insulin-Like Growth Factor Pathway in Rhabdomyosarcomas: Rationale and Future Perspectives

**DOI:** 10.1155/2011/209736

**Published:** 2011-03-03

**Authors:** Ana Sofia Martins, David Olmos, Edoardo Missiaglia, Janet Shipley

**Affiliations:** ^1^Molecular Cytogenetics, The Institute of Cancer Research, 15 Cotswold Road Sutton, Surrey SM2 5NG, UK; ^2^Sarcoma Unit, The Royal Marsden NHS Foundation Trust, London SW3 6JJ, UK; ^3^Bioinformatics Core Facility, Swiss Institute of Bioinformatics, 1015 Laussane, Switzerland

## Abstract

Rhabdomyosarcomas (RMS) are a heterogeneous group of tumors that share features of skeletal myogenesis and represent the most common pediatric soft tissue sarcoma. Even though significant advances have been achieved in RMS treatment, prognosis remains very poor for many patients. Several elements of the Insulin-like Growth Factor (IGF) pathway are involved in sarcomas, including RMS. The IGF2 ligand is highly expressed in most, if not all, RMS, and frequent overexpression of the receptor IGF1R is also found. This is confirmed here through mining expression profiling data of a large series of RMS samples. IGF signaling is implicated in the genesis, growth, proliferation, and metastasis of RMS. Blockade of this pathway is therefore a potential therapeutic strategy for the treatment of RMS. In this paper we examine the biological rationale for targeting the IGF pathway in RMS as well as the current associated preclinical and clinical experience.

## 1. Introduction

Rhabdomyosarcomas (RMS) are the most common soft tissue sarcoma of childhood [1, 2] with an incidence of 4.5 cases per million children/adolescents per year in the United States [3]. They are divided in two main histological variants: Embryonal (ERMS, 60–70% of all RMS cases) and Alveolar (ARMS, approximately 30%). Other minor variants include botryoid RMS, considered a subgroup of ERMS, and pleomorphic RMS, that occur in adults [1, 2, 4]. ERMS are predominant in younger patients and are generally associated with a good outcome in nonmetastatic cases, while ARMS are considered to be a tumor of adolescents and young adults that generally have a worse prognosis [2, 3, 5, 6]. The majority of ARMS are characterized by specific translocations between the DNA binding encoding domain of either the *PAX3* or *PAX7* genes and the transactivation encoding domain of *FOXO1* [7–9]. Rare variants involve fusion of the *PAX3* gene to members of the nuclear receptor transcriptional coactivator family of genes [10]. An estimated 30% of all histopathologically defined ARMS do not have these fusion transcripts [11] and recent gene expression profiling studies have indicated that these tumors biologically and clinically are more similar to ERMS than fusion gene positive ARMS [12, 13]. Other genetic events are associated with these tumors including those considered to cooperate with the fusion gene product in ARMS such as *MYCN* amplification and overexpression, and mutation of *TP53* [14–19]. ERMS are not characterized by specific fusion genes but are aneuploid with frequent gain of chromosome 8 and have activating mutations of *RAS* genes [20, 21]. Another frequent genetic alteration present in RMS is loss of heterozygosity (LOH) at the 11p15.5 locus. The region includes the genes *IGF2*, *H19*, and *CDKN11C* that are all subject to parental imprinting which can be aberrant in RMS and result in loss of imprinting (LOI) [22, 23]. In both ARMS and ERMS loss of heterozygosity or imprinting is thought to lead to overexpression of the gene encoding the insulin-like growth factor 2 (IGF2). Furthermore, overexpression of a receptor for this growth factor, IGF1R, is frequently found in RMS, occasionally associated with genomic amplification events [24]. Evidence supports IGF1R signaling in the genesis, growth, proliferation and metastatic behavior of RMS [25–27]. As the prognosis of RMS patients with metastatic or recurrent disease is still very poor, with only 30–40% achieving a cure, there is an urgent need to develop better therapies to treat these patients. In this paper we describe the evidence that implicates components of the IGF pathway in RMS development and examine the biological rationale for therapeutically targeting this pathway. We also consider the current preclinical and clinical experience with targeted approaches for treating RMS and suggest potential improvements that may be possible with combination strategies.

## 2. IGF Signaling in RMS

**Figure 1:**
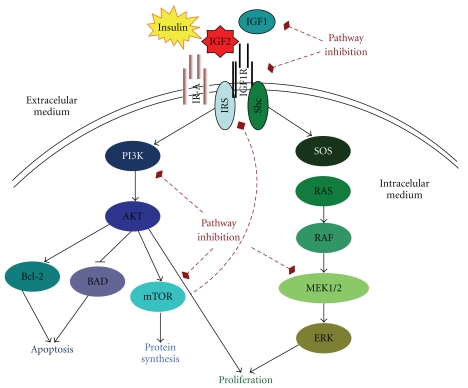
A simple schema of the IGF pathway and approaches to its inhibition. Insulin, IGF2 and IGF1 bind to their specific receptors including IGF1R, IGF2R, IR and hybrid receptors. Ligand binding results in the autophosphorylation of the tyrosine residues on each receptor, leading to recruitment of the adaptor proteins IRS and Shc to the receptor *β*-subunits intracellular domains. This process activates different signaling cascades through the PI3K-AKT and the RAS/RAF/MEK/ERK pathways resulting in stimulation of translation and cell cycle progression, increased proliferation and growth and inhibition of apoptosis. The dashed arrows indicate potential feedback mechanisms and points for strategic intervention to inhibit IGF1R signaling using anti-IGF1R mAbs or tyrosine kinase inhibitors (TKIs). Relevant downstream intracellular tyrosine kinase proteins to inhibit include PI3K, AKT, RAF, MEK and mTOR.

Components of the IGF pathway consist of 3 ligand molecules (IGF1, IGF2 and insulin), 6 binding proteins (IGFBP1 through to IGFBP6), and 4 receptors (IGF1R, IGF2R, IR and hybrid receptors). These orchestrate a cascade of signals (Figure 1) involved in numerous developmental and mitogenic pathways that lead to cellular processes such as activation of cell proliferation, invasion, and angiogenesis as well as inhibition of apoptosis [28, 29]. IGF2 and IGF1R are two components of the signaling pathway that are known to play a significant role in RMS oncogenesis.

### 2.1. IGF2 in RMS

IGF2 is normally expressed in the liver and other extrahepatic sites, similar to IGF1. Unlike IGF1, IGF2 expression in mammals is not just regulated by growth hormone (GH). However, the mechanisms regulating IGF2 expression remain uncertain. IGF2 is the predominant circulating IGF, with plasma levels 3- to 7-fold higher than IGF1 [31, 32]. 

**Figure 2:**
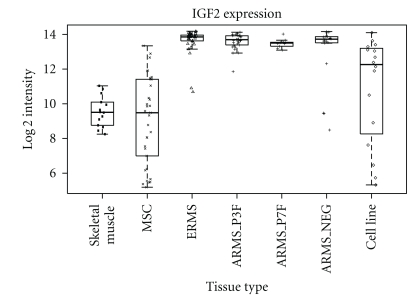
Levels of RNA expression for IGF2 derived from expression profiling data (Affymetrix HGU133plus2) in a panel of different tissues samples. These include normal skeletal muscle (Skeletal muscle), mesenchymal stem cells (MSC), ERMS, ARMS (*PAX3-FOXO1* and *PAX7-FOXO1* fusion positive, ARMS_P3F and ARMS_P7F and fusion gene negative ARMS_NEG) cases [13] and RMS cell lines (RH3, SCMC, RMS, RH30, RD, RMS-YM, RH18, Ruch3, T91-95, RH41, TE617T, Hs729T, T174, TE441T, Ruch2, and RH4) [30].

In RMS, several studies have shown overexpression of IGF2 in both cell lines and primary tumors [25, 33]. This is confirmed by our analysis of expression profiling data for a panel of RMS patient samples (Figure 2). LOH and LOI are the principal mechanisms underlying these IGF2 expression levels [22, 34]. In most nonmalignant tissues, *IGF2* is transcribed from the paternal allele, with the maternal allele being imprinted and consequently silenced by methylation. The imprinting of *IGF2* is influenced by the product of the downstream *H19* gene, with these two genes showing opposite imprinting patterns and transcription from *H19* occurring from the maternal allele. The process of LOI leads to biallelic expression (both paternal and maternal alleles) of the *IGF2* gene and IGF2 overexpression [23, 35]. LOH has been shown for ERMS in particular, with loss of the maternal 11p15.5 locus and duplication of the paternal *IGF2* allele (paternal isodisomy) that results in expression from the two paternal genes [36].

It has also been shown that increased IGF2 expression could be due to enhanced expression of transcriptional initiators such as AP-2 [37]. Potential AP-2-binding sites have been identified in the promoters of both the *IGF2* and *IGF1R* genes with an increase in AP-2-dependent *IGF2* mRNA expression found in RMS cases compared to normal skeletal muscle. In addition, loss of p53 has been shown to be associated with increased expression of IGF2 in RMS, even though the mechanisms supporting this are not fully elucidated [38]. The consistent overexpression of IGF2 in both ERMS and ARMS [25, 39] has led to the suggestion that IGF2 could be used as a marker for their differential diagnosis [25]. 

El-Badry and colleagues first demonstrated that IGF2 was acting as an autocrine and paracrine growth factor stimulating cell line growth and motility in RMS [27]. Later on, the same group investigated the potential of IGF2 to activate IGF1R and IGF2R and showed that the mitogenic response was primarily mediated though IGF1R [26].

Based on the fact that PAX3, PAX7, and IGF2 are involved in growth and differentiation, Wang and colleague's investigated the potential oncogenic cooperation between IGF2 and PAX3 or the PAX3-FOXO1 fusion protein. Mouse myoblasts transfected to express IGF2 alone or cotransfected to also express either PAX3 or PAX3-FOXO1 were transformed *in vitro* and could form tumors *in vivo* [40]. Only cells expressing both IGF2 and PAX3-FOXO1 developed invasive, poorly differentiated tumors with low rate of apoptosis. It has also been shown that the PAX3-FOXO1 fusion protein can induce both IGF2 and IGF1R expression that results in enhanced IGF signalling [41, 42].

IGF2 appears to be consistently overexpressed and acts as an autocrine/paracrine growth factor signaling through IGF1R in RMS. Its likely key role in the development and progression of both ARMS and ERMS is consistent with therapeutically targeting this pathway for the treatment of patients with RMS.

### 2.2. IGF1R in RMS

IGF1R is a transmembrane receptor with two extracellular ligand-binding *α*-subunits and two *β*-subunits forming the transmembrane and tyrosine kinase catalytic domains that are linked by disulfide bonds. It is primarily activated by its cognate ligands, IGF1 and IGF2 (IGF2 with 2- to 15-fold lower affinity) and by insulin with a lower affinity [28, 43–45]. The binding of the ligands to the cysteine-rich domain of the *α*-subunits leads to a conformational change of the *β*-subunit, stimulating the tyrosine kinase activity. This is followed by autophosphorylation of a cluster of tyrosine residues on the *β*-subunits of the intracellular domains. Subsequently, insulin receptor substrates (IRSs) 1 to 4 and the Src homology collagen-like adaptor proteins (Shc) bind to the juxtamembrane domain of the *β*-subunit, initiating alternative intracellular signaling cascades [46–48]. One of these pathways leads to PI3K-AKT-mTOR activation, while another results in MAPKs (Mitogen-Activated Protein Kinases) activation (Figure 1). Depending on the cellular context, the activation of these pathways results in cell proliferation, protein synthesis, and/or inhibition of apoptosis. IGF1R signaling can also lead to disregulation of cellular adhesion and motility, and the stimulation of myogenic differentiation in RMS [26, 27, 49, 50].

**Figure 3:**
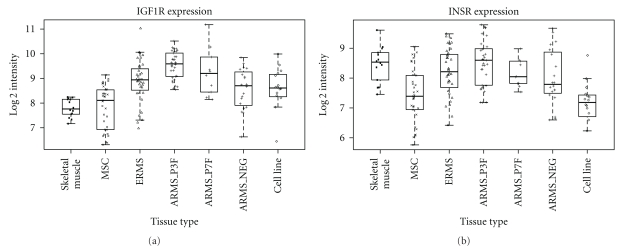
Levels of RNA expression of IGF1R and INSR (both isoforms of the insulin receptor combined) derived from Affymetrix HGU133plus2 expression profiling data of a panel of different tissues samples. These include normal muscle (Skeletal muscle), mesenchymal stem cells (MSC), ERMS, ARMS (*PAX3-FOXO1* and *PAX7-FOXO1* fusion positive, ARMS_P3F and ARMS_P7F and fusion gene negative ARMS_NEG) cases [13] and RMS cell lines (RH3, SCMC, RMS, RH30, RD, RMS-YM, RH18, Ruch3, T91-95, RH41, TE617T, Hs729T, T174, TE441T, Ruch2, and RH4) [30].

Both RMS tumors and cell lines express IGF1R [27], with IGF1R protein detected in more than 80% of all RMS cases without significant differences between ARMS and ERMS [51]. This is consistent with expression at the RNA level in our analysis of primary RMS patient data (Figure 3). An elevated level of receptor expression has been found to be associated with inferior survival rates [52] and has been used as biomarker for response to targeting the pathway in RMS preclinical models [53]. In this work it has been shown that, even though IGF1R was expressed in almost all samples studied, there was a large variation in expression levels that correlated with different levels of dependence on IGF1R prosurvival signaling. This led to proposing the notion of addiction to IGF1R in some tumor cells.

### 2.3. IR-A in RMS

The Insulin Receptor (IR) and IGF1R have evolved from a common ancestral gene encoding proteins with related functions and a very similar tetrameric structure; 2 *α*-subunits containing ligand-binding domains and 2 *β*-subunits with tyrosine-kinase domains [54, 55]. Cells and tissues coexpress both receptors and hybrid receptors can be formed by one *α*- and one *β*-subunit IR heterodimer, and one *α*- and one *β*-subunit IGF1R heterodimer [56, 57]. Furthermore, IR has two different isoforms: IR-A (or fetal) and IR-B (classic), which are determined by alternative splicing mechanisms (IR-A lacks exon 11) [58, 59]. Even though IR-A expression in adult cells is much lower than IR-B, this is not the case for cancer cells [60], but the factors contributing to the switch from isoform B to A expression in cancer are poorly understood [59, 61]. Increased expression of IR-A has been reported in carcinomas of breast, colon, lung, thyroid, and ovary [59]. Similarly, an elevated level of IR-A expression has been seen in osteosarcoma [62] and leiomyosarcoma [63] cell lines although the situation in RMS is currently unknown. In addition, IR-A is frequently expressed in solitary fibrous tumors samples (whilst IGF1R is not usually detected) [64] and is essential for virus-induced malignant transformation in Kaposi's sarcoma [65]. 

Phosphorylated IR in RMS has been described *in vitro* [66]. An increase in tyrosine phosphorylation of the insulin receptor substrate-1 (IRS-1) has also been reported in RMS. In poor prognosis patients this IRS-1 activation seems refractory to a negative feedback loop mediated by increased phosphorylated mTOR and 70S6 levels [52] which are observed in normal cells and RMS with a favourable prognosis. Thus, these facts support a persistent activation of the IR-IGF1R-mediated survival signaling in RMS patients, which may contribute to a worse prognosis in this malignancy.

## 3. Targeting the IGF Pathway in RMS

IGF1R has been acknowledged as a biologically relevant target in pediatric sarcomas for some time, but it has been difficult to, target it therapeutically due to its similarity to the IR and the toxicities associated with nonspecific inhibition. Nevertheless, in the last few years, new agents have emerged and have shown promising results. Essentially, the strategies for blocking or disrupting IGF1R include (a) the reduction of ligand levels or bioactivity, (b) the inhibition of receptor function using receptor-specific antibodies or small-molecule tyrosine kinase inhibitors (TKIs), or (c) inhibition of its downstream signaling molecules [67].

### 3.1. Targeting the Ligands

The disruption of the hypothalamus-hypophysis axis, and thus the clinical inhibition of GH release, can result in a decrease of circulating levels of IGF. Thus the disruption of this axis has been proposed as a potential strategy to reduce IGF in those cases where there is a background of elevated endocrine IGF release such as Beckwith-Wiedemann Syndrome which is associated with high rate of tumors in childhood, including RMS [68]. Another approach consists in reducing the concentrations of free active ligands using monoclonal antibodies against IGFs. DX-2647 is an antiligand monoclonal antibody which blocks IGF2, and also, but with less affinity, IGF1. Recently, this antibody has shown potential antitumor activity in human hepatocarcinomas xenografts [69], a tumor where upregulation of IGF2 expression is a common alteration. Even though there is not yet data available in sarcomas, this seems a plausible option for investigation in RMS where IGF2 is commonly upregulated. Other novel strategies to lower the ligand bioactivity may include recombinant IGFBPs [70]. *In vivo* experiments using the RMS cell line RH30 have shown that IGFBP-6 overexpression resulted in a marked delay in tumor growth in nude mice [71]. IGFBP-6 is unique among other binding proteins because of its binding specificity for IGF2. IGF2 has a higher affinity for IGFBP-6 than for IGF1R [72] suggesting that IGFBP-6 can reduce the levels of free active IGF2, preventing its binding to the receptor.

### 3.2. Targeting IGF1R

At the time of this paper, mAbs against IGF1R represent the most tangible clinical option, but there are also numerous small molecule tyrosine kinase inhibitors (TKIs) against IGF1R currently undergoing clinical evaluation [73]. Some of these small molecules also inhibit IR-A [74]. 

RMS cell lines secreting IGF2 have been shown to be able to grow in serum-free media. Under the same conditions, treatment of these cells with an antibody against IGF1R significantly inhibited cell growth suggesting that IGF2 functions as an autocrine and paracrine growth factor in RMS [27]. 

Overall, inhibition ligand binding using competitive antibodies and TKI have both been shown to block IGF1R activity resulting in inhibition of RMS cell proliferation, increased apoptosis, and cell cycle arrest [75, 76]. Furthermore suppression of vasculogenesis has also been demonstrated *in vitro *and* in vivo *xenograft models [77].* In vivo*, tumor formation and growth of RMS cells was inhibited by treating mice with an antibody antagonistic against IGF1R [53, 78, 79] or with TKIs [75, 76, 80]. The most effective antibodies against IGF1R include *α*IR3, which detects the *α*-subunit of IGF1R [78], and IMCA-12 [81]. The latter has shown promising results in the Pediatric Preclinical Testing Program [81]. Regarding the TKIs, we can highlight NVP-AEW541 [75, 76] and BMS-754807 [80] as two promising molecules to move towards testing at the clinical level. 

Other approaches for investigating the role of IGF1R have also been optimized recently, including using antisense RNA to reduce levels of expression and expression of a kinase-deficient form of this receptor [82, 83]. Both approaches resulted in tumor suppression.

### 3.3. Targeting Pathways Downstream of IGF1R

Recently, it has been shown, both *in vitro* and *in vivo*, that IGF1R survival signaling in RMS is primarily maintained through the AKT pathway, and that effective disruption of the IGF1R survival signaling results in decreased AKT activation [84]. However, activation of the PI3K pathway downstream of IGF1R and IR is subject to a negative feedback loop by mTOR through inhibition of IRS1 [85] (Figure 1). This is especially important in view of the fact that the combination of an antibody targeting IGF1R combined with an mTOR inhibitor, such as rapamycin, is predicted to inhibit RMS cell growth more effectively than either agent used alone. Indeed, an increase in AKT activation was found in RMS cells after rapamycin treatment with a more efficient inhibition of RMS growth both *in vitro* and *in vivo* when combined with an IGF1R antagonistic antibody [86, 87]. It has been described that patients with an increased phosphorylation of AKT, that result from a disruption in the feedback mechanism between mTOR and IRS, have a poorer survival [52]. Preclinical studies have also recently shown that targeting MEK/ERK (using the MEK/ERK inhibitor U0126) also leads to growth arrest of RMS tumors in an *in vivo* xenograft model [88]. All of these results provide preclinical evidence to support the use of signal transduction-based targeting of AKT/MEK in strategies for treating RMS.

## 4. Clinical Targeting of IGF1R in RMS: Evidence and Trends

In recent years, several agents against IGF1R have entered clinical trials of various tumor types, including sarcomas and RMS. A small number of clinical responses in patients with sarcomas have been reported across the different phase I clinical trials using IGF1R antibodies [89–91] and have raised hope for the success of this therapeutic modality. However, objective radiological responses were generally limited to patients with Ewing's sarcoma [89–91], with occasional prolonged (>6 months) disease stabilisation and clinical benefit in other sarcomas subtypes [89]. To our knowledge, only 2 patients with RMS were enrolled in these early trials. Both cases were heavily pretreated metastatic ARMS and both progressed within 6 weeks of starting treatment on figitumumab (a monoclonal antibody against IGF1R) [89]. More recently, in a preliminary report of the SARC011, a phase II trial in multiple sarcoma types, described 3 objective radiological responses in patients with RMS treated with the anti-IGF1R antibody R1507 [92]. However, more mature data in Ewing's sarcoma has shown that many responses only lasted for a finite period of time [93, 94].

Despite the difficulties of drawing conclusions from small numbers of RMS patients treated with anti-IGF1R antibodies, it is plausible to suggest that such single agent therapy in RMS might be insufficient to cause a clinically significant and persistent disruption in the IGF-mediated survival signalling, as seen in other neoplasias where IGF2 plays a relevant role [95]. Some preclinical studies have indicated that there are different binding epitopes on IGF1R that have differing biological activities [96] and different antibodies with distinct mechanisms of action to these epitopes [97]. Furthermore, combination strategies focused on blocking both IGF1 and IGF2 with two different inhibitory antibodies which resulted in enhanced inhibition of intracellular signalling through the IGF1R axis *in vitro* and *in vivo*, when compared to the activity of either single antibody alone. This effect was even more evident at high ligand concentrations where efficacy of monotherapy was relatively reduced [98]. A similar effect could be achieved by small molecule TKIs although few are currently in clinical development [73]. However, only prolonged disease stabilisation is reported in sarcoma patients treated within the OSI 906 (a TKI) phase I trial, although RMS patients were not included [99].

The efficacy of clinical strategies targeting IGF1R alone in RMS may be compromised due to the potential of cells to bypass the requirement for IGF2. Recently, it has been shown that IGF2 signaling can directly promote carcinogenesis in transgenic pancreatic neuroendocrine xenograft (an IGF2 dependent model) through IR binding [100]. Thus, RMS clinical alternatives could include the inhibition of both IGF1R and IR, using TKIs such as OSI-906 with activity against both IGF1R and IR-A [99]. However, this would potentially result in a higher metabolic toxicity. 

An alternative approach is to inhibit the IGF1R/IR downstream signaling cascade with PI3K/AKT/mTOR and/or Raf/Ras/MEK/ERK inhibitors. There are several molecules against these targets that have been recently tested in patients with various tumor types. Some of these, as single or combinations of agents, are currently undergoing pivotal phase III trials for regulatory approval in solid tumors other than sarcoma [101, 102]. Many agents have shown an adequate toxicity profile in phase I dose-finding studies and phase II trials, but to date, the clinical results with novel drugs in sarcomas, and specifically RMS in children, are limited. The largest experience in sarcomas has been provided with the study of mTOR inhibitors, particularly with compounds similar to rapamycin such as ridaforolimus, everolimus, and temsirolimus. These have shown some activity in adult soft tissue sarcomas [103]. Combining inhibitors of IGF1R/IR downstream signaling cascades, such as mTOR inhibitors, with an inhibitor of IGF1R also represents an attractive approach. A preliminary phase I trial report for figitumumab in combination with the mTOR inhibitor everolimus has shown activity in various sarcomas, including solitary fibrous tumors [104], which are characterised by the expression and secretion of high molecular weight proforms of IGF2 (“big”-IGF2) [105, 106] and constitutive activation of IR-A but not IGF1R [64]. Similar trials that include RMS are either ongoing or planned. 

Another strategy to consider is decreasing the levels of bioactive ligands using anti-IGF antibodies. Reducing circulating IGF has been unsuccessfully with somatostain analogues such as octreotide [107]. Recently, a human recombinant GH receptor antagonist, called pegvisomant, has been successful in tests for the treatment of acromegaly [108]. This pegylated recombinant human analogue of GH can decrease production and release of both IGF ligands [109]. Neither octreotide nor pegvisomant would impact on the paracrine IGF2 levels when they are genetically upregulated within the tumor—which is the case in RMS, but there is epidemiological evidence to support a role of the GH-regulated IGFs secretion in the promotion, progression, and maintenance of tumors in childhood and adolescence. Currently, a phase I clinical trial of figitumumab in combination with pegvisomant (NCT00976508) [110] is active in adults patients with solid tumors, but it will also enroll patients 10 years or older with refractory sarcomas.

A final clinical strategy in RMS could be sequential or parallel IGF1R pathway blockade combined with inhibition of the Erb2 [53] or PDGFR*α* [111] axes, that are potentially involved in resistance to IGF1R therapies. These pathways in themselves may also be of therapeutic benefit to inhibit in some RMS [112–116]. One way to address the issue of controlling drug sensitivity, as well as pathway cross talk, is to control the response to stress response mechanisms associated with drug treatment. Heat shock stress is a cellular response to stress induced by drug treatment in which the cell increases the expression of several key molecules, called heat shock proteins (HSPs), in order to protect against the effects of treatment. HSPs are chaperone proteins that help to maintain protein stability, renature unfolded proteins, or target their degradation [117, 118]. Several of these HSP client proteins are involved in signal transduction pathways that lead to proliferation, apoptosis, or cell cycle progression in several cancers, which is precisely the case for IGF1R [119, 120]. Therefore, HSP inhibition is a therapeutic strategy to inhibit multiple receptor pathways. IGF1R chaperoning by HSP90 and its possible relationship with resistance to IGF1R targeting has been shown in Ewing's sarcoma. HSP90 was differentially expressed between Ewing's sarcoma cell lines sensitive versus resistant to treatment and HSP90 inhibition reduced Ewing's sarcoma cell line growth and survival, especially in the cell lines resistant to IGF1R inhibitors [121]. An analogous situation may be the case for RMS. It has been shown that HSP90 inhibitors, geldanamycin, and its analogs, can profoundly affect the proliferation of RMS cells, including inducing apoptosis and downregulating the expression of AKT [122].

## 5. Conclusions

There is a large amount of preclinical, clinical, and epidemiological data supporting targeting the IGF1R pathway in sarcomas, and specifically RMS. The activity of IGF1R monoclonal antibodies has been confirmed by the early reports of clinical activity in Ewing sarcoma [89–91, 94]. However, in RMS patients, despite some responses observed with R1507 [92], targeting IGF1R alone does not seem the optimal strategy due to the complexity of this pathway and the key role of IGF2 in this pathology. To extend the benefits of these therapeutic approaches there is an urgent need to identify predictive biomarkers to improve patient selection and facilitate the development of rational combination regimens. It is likely that a suite of biomarkers, both in the host and tumor [73] will be required rather than single biomarker selection, with some candidates for study in RMS including IGF2, pIGF1R/IGF1R, IGF1, pIRS-1/IRS-1, pIR-A/IR-A, IGFBP-6, and maybe others such as HSPs, PDGFR and Erb2.

##  Conflict of Interests

The authors have no conflict of interest to declare.
